# Assessment of intracranial aneurysm neck deformation after contour deployment

**DOI:** 10.1007/s11548-024-03189-w

**Published:** 2024-05-31

**Authors:** Lena Spitz, Jana Korte, Franziska Gaidzik, Naomi Larsen, Bernhard Preim, Sylvia Saalfeld

**Affiliations:** 1 Department of Simulation and Graphics, Otto-von-Guericke University, Universtitaetsplatz 2, 39106 Magdeburg, Sachsen-Anhalt Germany; 2 Research campus STIMULATE, Magdeburg, Germany; 3 Laboratory of Fluid Dynamics and Technical Flows, Otto-von-Guericke University, Magdeburg, Germany; 4 Department of Radiology and Neuroradiology, University Medical Center Schleswig-Holstein, Kiel, Germany; 5 Computational Medicine Group, Technical University Ilmenau, Ilmenau, Germany

**Keywords:** Intracranial aneurysm, Morphological analysis, Contour neurovascular system, Wide-necked bifurcation aneurysm

## Abstract

**Purpose:**

The contour neurovascular system (CNS) is a novel device to treat intracranial wide-necked bifurcation aneurysms, with few studies assessing its long-term effects. Particularly its impact on aneurysm morphology has not been explored yet. We present a preliminary study to explore this impact for the first time, focusing on the neck curve and ostium of the aneurysm.

**Methods:**

We investigated seven aneurysms treated with the CNS to assess ostium deformation after CNS deployment by comparing models extracted from in vivo medical pre-treatment and follow-up scans via morphological analysis. Time between pre- and follow-up scans was ten months on average. Size and shape indices like area, neck diameter, ellipticity index, undulation index, and more were assessed.

**Results:**

Ostium size was reduced after treatment. On average, ostium area was reduced at a rate of $-$0.58 (± 4.88) mm^2^ per year, from 15.52 (± 3.51) mm^2^ to 13.30 (± 2.27) mm^2^, and ostium width from 5.01 (± 0.54) mm to 4.49 (± 0.45) mm, with an average reduction of $-$0.59 (± 0.87) mm. This shrinking positively correlated with time passing. Shape deformation was low, though notably mean ellipticity index was reduced by 0.06 (± 0.15) on average, indicating ostia were less elongated after treatment.

**Conclusion:**

We interpret the shrinking of the ostium as part of the healing process. Shape changes were found to be small enough to conclude no shape deformation of the ostium from CNS deployment, but the analysis of more cases with more parameters and information is necessary.

## Introduction

Intracranial aneurysms (IAs) are pathological dilatations of the blood vessels in the brain that bear the risk of rupture. While rupture has catastrophic consequences with a mortality of up to 50% [1], less than 2% of IAs rupture in a patients’ lifetime [2]. For those with a high rupture risk, different treatment options are available, with endovascular treatment becoming the preferred treatment technique over surgical interventions [3].

Wide-necked bifurcation aneurysms (WNBAs) are a sub-type of IAs that are challenging to treat with conventional methods of endovascular treatment and surgical interventions. Complete occlusion is achieved in only 46% and adequate occlusion in 60% of cases, with a complication rate of 19% [4, 5].

**Fig. 1:**
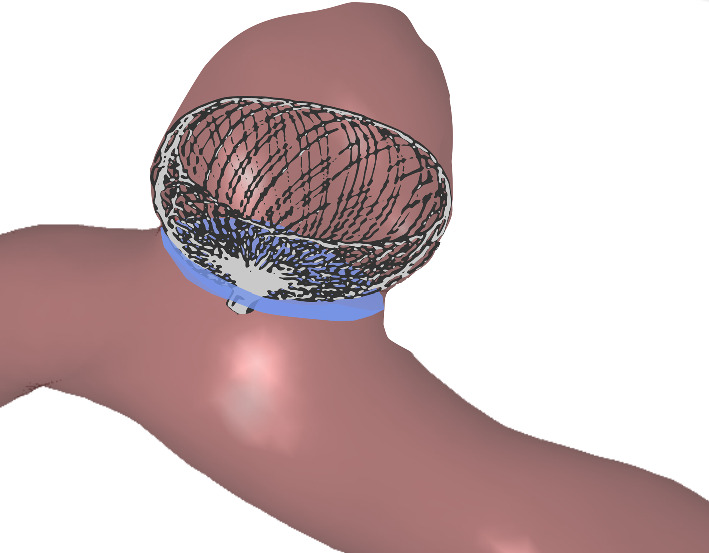
Example of marked ostium (blue) and CNS (gray) placement within an IA (red)

Thus, new treatment options were designed to address the specific challenges of WNBAs. The contour neurovascular system (CNS, Stryker, Kalamazoo, MI, USA) was recently developed: It is a self-expanding cup-shaped device consisting of braided wires which is implanted in the IA neck (Fig. 1), where it disrupts the blood flow into the IA and diverts it from the ostium. A comprehensive review of retrospective studies with 131 cases found it safe and efficient to use with a pooled adequate occlusion rate of 84% [6], including ruptured IAs [7–9]. In comparison with other intra-saccular flow disruptors, correct sizing of the CNS is presumably easier to achieve since only neck and dome diameter have to be considered, while aneurysm height does not influence the choice of size of the device [6–8]. While these studies are promising, more cases with longer follow-up studies are needed [6]. Furthermore, due to the novelty of the device, many long-term effects and recommended uses are not known or researched yet.

So far there is one study examining the effect of the CNS on the blood flow [10]. The authors performed an in silico and in vitro comparative study using magnetic resonance imaging (MRI) and computational fluid dynamics of WNBA phantoms and found that the CNS effectively reduces blood flow in the IA sack. They also found indications that CNS sizing has a greater impact on flow disruption and diversion properties than positioning.

There have been no studies published so far examining the effect of the CNS on IA morphology. Post-therapeutic changes of aneurysm morphology possibly influence the healing process and recurrence rates. This work serves as a first comparison of neck curves and ostia of IAs before and after treatment with the CNS.

## Materials and methods

Our data included seven IAs treated with the CNS. We compared in vivo scans taken pre-treatment (Pre) to follow-up (FU) 3D rotational angiographic images (3D RA) on an Allura Xper FD 20/10 (Philips, Best, The Netherlands), all with a spatial resolution of 384$\times$384$\times$384 and an isotropic voxel size ranging from 0.16 to 0.33 mm after reconstruction. The time between Pre and FU scans ranged from two months and three weeks to two years and two months, with a mean of 10 months and two weeks.

All IA images were segmented into 3D models [11], with the approach extended to work for 3D RA by adjusting pre-processing parameters manually. The models were then manually cut to the region of interest comprising mainly the IA, and ostia were extracted automatically [12]. This was possible for all Pre datasets and for three of the FU datasets. For the other four FU datasets, the aneurysm sack was not visible after treatment and automatic extraction failed; thus, the ostium was extracted manually.

**Fig. 2:**
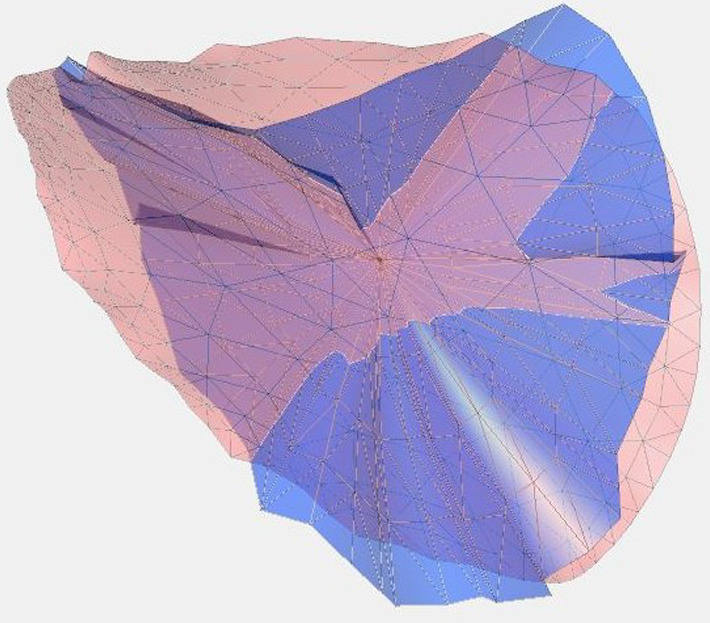
Example of registered pre-treatment (pink) and follow-up (blue) ostia models

A global rigid iterative closest point registration was used to align the Pre and FU models (IAs and surrounding vasculature) of each case, which automatically registered the ostia (Fig. 2). Automatic registration failed for one case, which was then manually registered, globally and rigidly. For qualitative deformation analysis of the ostia, we utilized arrow visualizations based on correspondences and minimal distances to illustrate areas of low and strong deformations. For quantitative analysis, we used morphological parameters, namely size and shape indices, the first describing size of the morphology and the latter size-independent shape that relates to ellipticity and concavity of the ostia. Derivation of all parameters was implemented in MATLAB 2023a (The MathWorks Inc., Natick, MA, USA), or they were extracted with its internal functions.

Size indices included area, diameter, and convex hull (CH) volume, as well as distance between the registered ostia as calculated by the symmetric surface distance (SSD). The maximum diameter describes an ostium’s width and would determine the CNS’s sizing.

Shape indices included ellipticity index (EI), undulation index (UI), and non-sphericity index (NSI), as defined by [13]. We also calculated mean and Gaussian curvature of the ostium surface. Elongation of a shape is described by the EI that characterizes the fit of a shape into a hemisphere. It does not relate information about undulation or convexity of the shape, as it is calculated based on the CH volume $V_{CH}$ and area $A_{CH}$ [13]:

1 $$\begin{aligned} EI = 1 - (18\pi )^{\frac{1}{3}} \frac{V_{CH}^{\frac{2}{3}}}{A_{CH}} \end{aligned}$$

UI measures the convexity of a shape by comparing volume *V* of the shape to $V_{CH}$, and NSI measures how close a shape is to a perfect sphere based on *V* and area *A* [13]:

2 $$\begin{aligned} UI= & {} 1 - \frac{V}{V_{CH}} \end{aligned}$$

3 $$\begin{aligned} NSI= & {} 1 - (18\pi )^{\frac{1}{3}} \frac{V^\frac{2}{3}}{A} \end{aligned}$$

As the ostium is a surface and does not have a volume, we used surface adaptations of UI and NSI [14]. Instead of volume *V*, they utilize area *A* and circumference *C*, and the NSI thus becomes the Non-circularity Index (NCI) [14]:

4 $$\begin{aligned} UI_{2D}= & {} 1 - \frac{A}{A_{CH}} \end{aligned}$$

5 $$\begin{aligned} NCI= & {} 1 - 2 \sqrt{\pi } \frac{A^\frac{1}{2}}{C} \end{aligned}$$

where necessary, we projected the ostia onto a 2D plane based on principal component analysis for minimum information loss. Low values of the indices close to 0 describe a shape close to a perfect circle, while high values close to 1 describe high ellipticity or concaveness.

Rate of change was calculated by dividing absolute change ($\Delta$) of a parameter by the number of days between Pre and FU scans, and multiplying the daily rate of change by 365 to reach rate of change per year ($\frac{\Delta }{year}$).

We did not assess deformations of the entire aneurysm sack, because the IA dome is not visible in the underlying angiographic data as the contrast agent does not uniformly opacify the IA sack after CNS deployment.

## Results

Comparing the size indices (Table 1), Pre ostia had an average area of 15.52 (± 3.51) mm^2^, with values ranging from 9.14 to 20.61 mm^2^, and FU ostia had an average area of 13.30 (± 2.27) mm^2^, ranging from 10.18 to 16.55 mm^2^. Three out of seven ostia were larger after treatment (Table 2), but on average, ostium area of the FU data was 10% smaller than Pre data, with an average absolute shrinkage of 2.22 (± 4.22) mm^2^ and an average yearly shrinkage rate of 0.58 (± 4.88) mm^2^, which makes -2 (± 0.26) % per year.

Pre ostia had an average width of 5.01 (± 0.54) mm and FU ostia 4.49 (± 0.45) mm, with an average difference of 0.51 (± 0.68) mm, making FU ostia width 9% smaller than that of Pre ostia. This mirrors results for mean diameter, where average difference was 0.39 (± 0.62) mm, and FU had a 8% smaller mean diameter on average. For yearly rate of difference, max diameter had one of $-$0.59 (± 0.87) mm, making -12 (± 19) %, and mean diameter one of $-$0.24 (± 0.78) mm, making -4 (± 18) %.

**Fig. 3:**
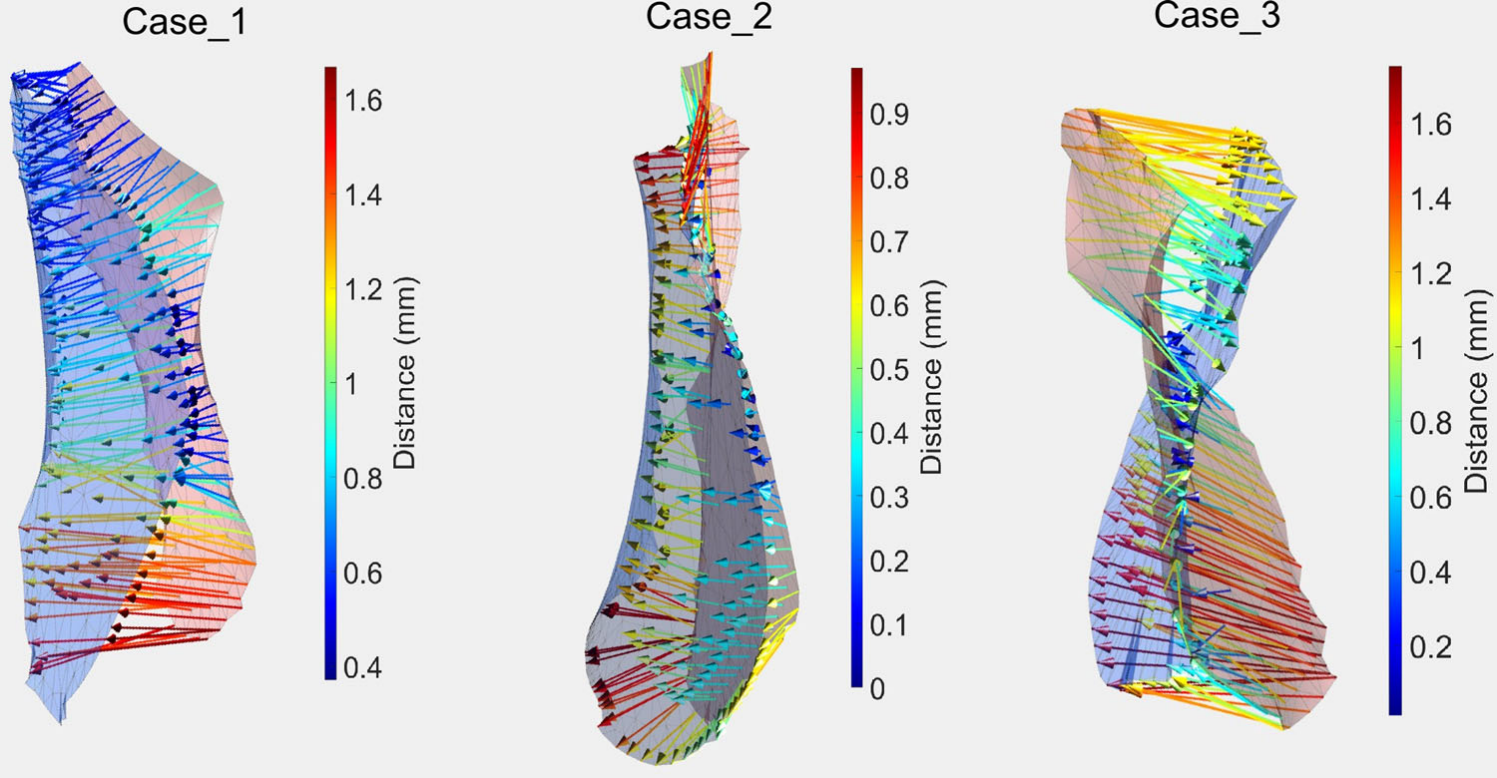
Example of the deformation of the ostia of three ostia from Pre (pink) to FU (blue) scans. Color-coded arrows pointing from Pre to FU data highlight areas of low (blue) and high (red) deformations

Distances denoted by mean SSD between the Pre and FU ostia ranged from 0.35 mm to 1.69 mm, with a mean of 0.74 (± 0.42) mm. Strength of deformation from Pre to FU ostia varied between individual cases, where some cases had a maximum deformation of 2.32 mm, and others had one of 0.78 mm (Fig. 3). Deformation seemed to be biggest around the edges of the neck curve and smaller in the middle of the ostium.

**Table 1:** Mean and standard deviation ($\sigma$) of all calculated parameters, as well as difference ($\Delta$) and rate of change per year ($\frac{\Delta }{year}$), for pre-treatment and FU ostia, as averages of all seven cases

	Pre		FU		$\Delta$		$\frac{\Delta }{year}$	
Parameter	Mean	$\sigma$	Mean	$\sigma$	Mean	$\sigma$	Mean	$\sigma$
SSD (mm)	0.74	0.42	–	–	–	–	–	–
Area (mm^2^)	15.52	3.51	13.30	2.27	2.22	4.22	$-$0.58	4.88
Max diameter(mm)	5.01	0.54	4.49	0.45	0.51	0.68	$-$0.59	0.87
Mean diameter (mm)	4.35	0.46	3.96	0.40	0.39	0.62	$-$0.24	0.78
CH volume (mm^3^)	6.81	2.41	6.38	1.83	0.43	3.08	1.11	3.02
EI	0.59	0.02	0.54	0.07	0.05	0.08	$-$0.06	0.15
2D UI_2D_	0.01	0.01	0.03	0.02	$-$0.02	0.03	0.01	0.04
3D UI_2D_	0.53	0.01	0.53	0.01	0.00	0.01	0.00	0.02
2D NCI	0.04	0.02	0.06	0.04	$-$0.03	0.06	0.00	0.10
3D NCI	0.08	0.02	0.10	0.04	$-$0.02	0.06	0.00	0.08
Mean curvature	0.02	0.03	0.00	0.08	0.02	0.06	$-$0.01	0.08
Gaussian curvature	$-$0.09	0.07	$-$0.16	0.13	0.08	0.17	$-$0.26	0.64

Correlating the time passed between the Pre and FU scans with the rate of change, we found a positive correlation between time passed and changes in area, width, and mean diameter. The Pearson correlation coefficient (PCC) for time and area change was 0.80 and had a *p*-value of 0.03, making it the only correlation with *p*-value < 0.05. PCC for width and mean diameter was 0.68 and 0.69, respectively.

As ostia are complex shapes that can be concave, we started the comparison of shape indices with the CHs. Here, we observed an average absolute increase in volume of 0.43 (± 3.08) mm^3^ and a rate of 1.11 (± 3.02) mm^3^ per year. However, when looking at average relations between Pre and FU scans, CHs of FU ostia were 4% larger. Average CH volume was 6.81 (± 2.41) mm^3^ and 6.38 (± 1.83) mm^3^ for Pre and FU ostia, respectively.

**Table 2:** All observations of parameters for all cases, including differences between Pre and FU data ($\Delta$) and rate of change per year ($\frac{\Delta }{year}$)

	Case 1		Case 2		Case 3		Case 4		Case 5		Case 6		Case 7	
Parameter	Pre	FU	Pre	FU	Pre	FU	Pre	FU	Pre	FU	Pre	FU	Pre	FU
Area (mm^2^)	13.47	16.42	16.31	12.40	15.50	16.55	19.22	12.59	9.14	10.18	14.40	13.74	20.61	11.19
Max diameter(mm)	4.78	5.29	4.70	4.07	5.24	4.85	5.74	4.66	4.37	3.99	4.42	4.51	5.78	4.07
Mean diameter (mm)	4.07	4.59	4.53	3.76	4.45	4.44	4.82	4.00	3.46	3.54	4.22	3.94	4.93	3.46
CH Volume (mm^3^)	5.43	8.24	6.39	8.41	6.73	7.09	10.05	4.15	3.24	3.25	5.39	7.23	10.47	6.31
EI	0.59	0.55	0.61	0.42	0.59	0.60	0.56	0.62	0.57	0.61	0.61	0.50	0.58	0.48
2D UI_2D_	0.02	0.01	0.00	0.04	0.01	0.02	0.01	0.01	0.02	0.01	0.00	0.03	0.01	0.08
3D UI_2D_	0.54	0.53	0.52	0.55	0.53	0.53	0.53	0.52	0.53	0.53	0.52	0.52	0.53	0.55
2D NCI	0.05	0.03	0.01	0.07	0.05	0.03	0.03	0.04	0.08	0.04	0.01	0.07	0.02	0.16
3D NCI	0.11	0.07	0.06	0.11	0.11	0.08	0.07	0.08	0.10	0.07	0.04	0.11	0.06	0.19
Mean curvature	0.03	0.02	$-$0.02	$-$0.10	0.01	0.03	0.02	0.04	0.08	0.11	0.01	0.02	0.02	$-$0.13
Gaussian curvature	$-$0.26	$-$0.05	$-$0.05	$-$0.12	$-$0.11	$-$0.21	$-$0.05	$-$0.02	$-$0.06	$-$0.45	$-$0.03	$-$0.09	$-$0.04	$-$0.21

	$\Delta$	$\frac{\Delta }{year}$	$\Delta$	$\frac{\Delta }{year}$	$\Delta$	$\frac{\Delta }{year}$	$\Delta$	$\frac{\Delta }{year}$	$\Delta$	$\frac{\Delta }{year}$	$\Delta$	$\frac{\Delta }{year}$	$\Delta$	$\frac{\Delta }{year}$
Days	170	–	196	–	165	–	802	–	81	–	263	–	554	–
Area (mm^2^)	$-$2.95	6.34	3.91	$-$7.29	$-$1.05	2.33	6.63	$-$3.02	$-$1.04	4.68	0.66	$-$0.91	9.42	$-$6.21
Max diameter(mm)	$-$0.51	1.09	0.63	$-$1.18	0.39	$-$0.86	1.08	$-$0.49	0.38	$-$1.72	-0-09	0.13	1.71	$-$1.12
Mean diameter (mm)	$-$0.51	1.10	0.77	$-$1.44	0.01	$-$0.02	0.82	$-$0.37	$-$0.08	0.35	0.26	$-$0.35	1.47	$-$0.97
CH Volume (mm^3^)	$-$2.81	6.04	$-$2.02	3.76	$-$0.35	0.87	5.89	$-$2.68	$-$0.01	0.06	$-$1.84	2.55	4.16	$-$2.74
EI	0.04	$-$0.09	0.19	$-$0.36	$-$0.01	0.03	$-$0.06	0.03	$-$0.04	0.17	0.11	$-$0.15	0.10	$-$0.07
2D UI_2D_	0.01	$-$0.03	$-$0.03	0.06	0.00	0.01	0.00	0.00	0.01	$-$0.04	$-$0.03	0.04	$-$0.08	0.05
3D UI_2D_	0.01	$-$0.02	$-$0.02	0.04	0.00	0.00	0.01	0.00	0.01	$-$0.02	0.00	0.00	$-$0.03	0.02
2D NCI	0.02	$-$0.04	$-$0.06	0.10	0.01	$-$0.03	$-$0.01	0.01	0.04	$-$0.19	$-$0.06	0.09	$-$0.13	0.09
3D NCI	0.04	$-$0.08	$-$0.05	0.10	0.03	$-$0.06	0.00	0.00	0.02	$-$0.11	$-$0.07	0.10	$-$0.12	0.08
Mean curvature	0.01	$-$0.02	0.08	$-$0.14	$-$0.02	0.03	$-$0.03	0.01	$-$0.03	0.12	$-$0.01	0.01	0.15	$-$0.10
Gaussian curvature	$-$0.20	0.44	0.07	$-$0.13	0.09	$-$0.21	$-$0.03	0.02	0.39	$-$1.75	0.07	$-$0.10	0.17	$-$0.11

The Pre ostia EI was higher than FU ostia EI in four out of seven cases (Table 2), with respective average EIs of 0.59 (± 0.02) and 0.54 (± 0.07) (Table 1). On average, the Pre EI was 8% larger than the FU EI, with a yearly rate of change of $-$0.06 (± 0.15), which is −10 (± 26) % per year. This means that ostia were more elongated before treatment with the CNS.

We applied the 2D-adapted formula for UI_2D_ (Eq. 4) to both the original ostium data and the 2D-projected data, as the latter would lose information about convexity in the z-dimension. For projected ostia, both Pre and FU ostia had small values, 0.01 (± 0.01) and 0.03 (± 0.02), respectively, meaning they were overall convex shapes with low changes after treatment. The original ostia had a nearly identical UI_2D_ of 0.53 (± 0.01), with an average difference of 0.004 and FU ostia being 0.7% less convex on average.

The projected Pre ostia had an average NCI of 0.04 (± 0.02), and FU ostia 0.06 (± 0.04). The non-projected ostia showed a similar trend, with Pre ostia having an average NCI of 0.08 (± 0.02) and FU ostia having one of 0.10 (± 0.04). These differences seem small, but when put in relation, the NCI of projected FU ostia was three times larger on average than for Pre ostia and the 3D NCI 52% larger. Overall, both Pre and FU ostia’s NCI is close to 0 and therefore rather circular, but slightly less so for FU ostia.

Lastly, we assessed mean and Gaussian curvature of the ostia. Pre ostias’ mean curvature was 0.02, and FU was 0.00. While an average difference of 0.02 (± 0.06) is small, the differences in individual ostium curvature from Pre to FU ostia go up to seven times larger. Gaussian curvature was $-$0.09 (± 0.07) for Pre ostia and $-$0.16 (± 0.13) for FU ostia on average, with an average difference of 0.08 (± 0.17), and FU observations being three times larger.

## Discussion

With an average SSD of 0.74 mm, which is 15% of average ostium width, changes in ostia after treatment with the CNS are subtle, but present. SSD compares vertices on the ostia surface mesh, and a meshing-independent distance measure could yield further information in future studies.

We found that on average, ostia were smaller after treatment in comparison to pre-treatment, as reflected by their area and diameters (Table 1). We also found a high positive correlation (PCC 0.80) between time passed between Pre and FU scans and shrinking of the ostium area, with a *p*-value of 0.03, pointing toward a significant correlation. This is supported by the yearly shrinkage rate of 0.58 (± 4.88) mm^2^, which is 2% area shrinkage per year on average. Shrinking of width and mean diameter showed a moderately positive correlation with time passed as well, but no statistical significance. Our small case number needs to be kept in mind, however. The shrinking of the ostia could be related to the healing process of the aneurysm after treatment, possibly including advancing fibrosis which has been described to be a feature of aneurysm healing [15], one of the goals of the CNS [8].

However, we also observed that on average, CH volume increased by 4%. At the same time, CH volume was, on average, 0.43 mm^3^ smaller for FU data. This is likely due to the individual cases. Five of seven cases were larger after treatment, but their average growth (1.41 mm^3^) was overall less than the other two cases’ average shrinking (5.03 mm^3^).

This might be due to shape changes after treatment. We found FU ostia to be slightly less elongated than Pre ones, based on the average EI decreasing from 0.59 to 0.54, which makes a shrinkage of 10% per year. UI_2D_ and NCI meanwhile increased, meaning after treatment, ostia were more concave and less circular. This increase, however, was very small, with yearly rates of change not above 0.01. 2D-projected ostia were overall very convex, Pre ostia UI_2D_ being on average 0.01 and 0.03 for FU ostia. Despite such small changes, UI_2D_ increased five times from Pre to FU on average. For the non-2D-projected UI_2D_, changes in Pre and FU were minuscule. The concavity, however, was higher in 3D overall, averaging at 0.53 for both Pre and FU, confirming that information loss through projection is noteworthy. We conclude that ostia are in general quite concave and, only by projecting to their principal component, become convex. NCI achieved similar results, with a small absolute increase from Pre to FU ostia (as little as 0.03), but a high increase in relation (up to three times larger). This supports the increase in curvature, with a three time increase from Pre to FU ostia.

The cases with the highest EI and NCI pre-treatment also had the highest reduction rate of the respective index (see Table 2), but there were several cases where EI and NCI increased. Interestingly, in case of NCI, four out of seven cases were less circular after treatment, on average three times less circular. Intuitively, one would expect increasing circularity after implantation of the circular CNS. The absolute changes in NCI were small, however, with 0.03 on average, and EI only increased by 0.05 on average.

The results of this study hint at complex interactions between the device and the aneurysm wall and their respective mechanical behaviors. With a sample size of seven cases, we can, however, not make any statements about statistical significances.

## Conclusion

While we cannot make a decisive statement about the change of the ostium shape after treatment with the CNS, we found that generally ostia sizes reduced over time according to size indices, with stronger shrinking as more time passed. This might enable faster endothelialization of the ostium and promote aneurysm healing [15]. We hypothesize this shrinking is related to the healing process of the vessel wall. We did not assess morphologic changes of the aneurysm sac in this study, which may complement the analysis of overall post-treatment changes in aneurysm morphology.

The results of this study provide valuable first insights into morphologic changes in IAs after CNS implantation which could be further used in hemodynamic and clinical studies to investigate their clinical impact.
